# High-fluorescent cells as a rapid screening tool for malignant effusions

**DOI:** 10.7717/peerj.20528

**Published:** 2025-12-18

**Authors:** Hongmei Ding, Yuexinzi Jin, Chu Chu, Lin Wang

**Affiliations:** 1 Department of Laboratory Medicine, The First Affiliated Hospital with Nanjing Medical University, Nanjing, China; 2 Branch of National Clinical Research Center for Laboratory Medicine, Nanjing, China

**Keywords:** Serous effusion, High-fluorescent cells, Carcinoembryonic antigen, Diagnostic performance, Lung cancer

## Abstract

**Objective:**

Elevated levels of high-fluorescent cells (HFCs) in serous effusions often suggest the presence of tumor cells. The purpose of this study was to evaluate the diagnostic value of HFC detection in the differentiation between benign and malignant serous effusions using the Sysmex XN-10 automated hematology analyzer in body fluid mode (BF mode).

**Methods:**

Serous effusion specimens, including 702 pleural effusions, 255 ascitic fluid samples and 21 pericardial effusions, were collected from 978 patients at the First Affiliated Hospital with Nanjing Medical University between June 2023 and June 2024. The absolute number (HFC#) and percentage (HFC%) of HFCs were detected using the Sysmex XN-10 automated hematology analyzer. Meanwhile, levels of carcinoembryonic antigen (CEA), lactate dehydrogenase (LDH) and other biomarkers in serous effusions were measured. The diagnostic performance for malignant effusions was evaluated using receiver operating characteristic (ROC) curves.

**Results:**

The HFC#, HFC% and CEA levels in the malignant effusion group were significantly higher than those in the benign effusion group (all *P* < 0.001). Multivariate logistic regression analysis revealed that HFC#, HFC%, CEA, and LDH were independently associated with malignant effusion. Receiver operating characteristic (ROC) analysis showed that CEA had the best diagnostic performance (AUC = 0.817), followed by HFC% (AUC = 0.727) and HFC# (AUC = 0.703). The diagnostic performance of HFC in combination with CEA is significantly better than that of CEA alone. For malignant effusions associated with lung cancer, the diagnostic performance of CEA was better than HFC and cytokeratin 19 fragment (CYFRA21-1), but there was no significant difference between HFC and CYFRA21-1.

**Conclusion:**

HFC demonstrates high diagnostic value in identifying malignant serous effusions, especially when used in combination with CEA. As a rapid laboratory parameter based on cellular nucleic acid characteristics, HFC can serve as a useful auxiliary tool for screening malignant effusions.

## Introduction

Serous effusion refers to the abnormal accumulation of fluid in the pleural cavity, abdominal cavity, or pericardial cavity under pathological conditions (Davidson, 2004) and can be caused by infection, tumors, trauma, and other conditions such as cardiac or hepatic diseases (Biggins et al., 2021; Lazaros et al., 2023). Rapid identification of the nature of the effusion is important for the diagnosis and treatment of diseases. In addition to routine examination, laboratory analysis of serous effusions includes cytological, biochemical, microbiological, immunological, and genetic evaluations. In particular, exfoliative cytology remains the gold standard for the diagnosis of malignant effusion (Bibby et al., 2018). However, this method is time-consuming, has limited sensitivity despite high specificity (Ahuja et al., 2024), and is susceptible to subjective interpretation by technicians (Shidham, 2021; Geyer, 2010; Thakur et al., 2022).

In recent years, a growing number of biomarkers have been used in the differential diagnosis of malignant serous effusion. Carcinoembryonic antigen (CEA) is a widely used glycoprotein tumor marker in clinical practice (Hammarström, 1999). Elevated CEA levels are primarily associated with various malignancies, particularly colorectal, lung, breast and gastric cancers (Jansen et al., 2024). While CEA demonstrates high specificity for malignant effusions, its sensitivity can be variable (Shi et al., 2008; Cheng et al., 2022; Yang et al., 2025). Additionally, non-malignant conditions such as inflammation, liver disease, and smoking can elevate CEA levels (Moretto et al., 2021).

The Sysmex XN series automated hematology analyzers can work in body fluid mode (BF mode) to count the cells in serous effusion samples and provide parameters including white blood cell count (WBC), red blood cell count (RBC), white blood cell differentials, and high-fluorescence cells (HFCs). The absolute number of HFCs (HFC#) and percentage of HFCs (HFC%) have received attention for their diagnostic value in malignant effusions (Zhang et al., 2025). Studies indicate that fluorescence intensity increases following nucleic acid staining due to infinite proliferation of tumor cells (Larruzea et al., 2018; Zimmermann et al., 2013), and HFC detection demonstrates high sensitivity (87%–93%) for identifying malignant effusions (Favresse et al., 2020; Cho et al., 2022). Therefore, elevated HFC levels may suggest the presence of tumor cells.

The purpose of this study was to assess the diagnostic value of HFCs in differentiating between benign and malignant serous effusions by using the BF mode of Sysmex XN-10 automated hematology analyzer.

## Materials & Methods

### Serous effusion samples collection

In this study, serous effusion samples were collected from 978 patients admitted to the First Affiliated Hospital with Nanjing Medical University between June 2023 and June 2024. A total of 621 male patients and 357 female patients were included in the study, with an age range of 13–99 years (median 67 years). Of the 978 serous effusion samples, 702 were pleural effusions, 255 were ascitic fluid samples, and 21 were pericardial effusions. All samples were collected in sterile tubes without anticoagulant and evenly divided into at least three aliquots for cell counting, exfoliative cytology, and other biomarker analysis. All analyses were completed within 2 h after collection. To reduce confirmation bias, cytological evaluations were performed blinded to HFC results. All testing procedures were conducted independently. Malignant effusion was diagnosed if any of the following criteria were met: (a) exfoliated cytology examination revealing malignant cells; (b) histopathological confirmation of malignancy by surgical or biopsy specimens; or (c) comprehensive assessment including imaging, clinical symptoms, effusion characteristics, tumor biomarkers, and follow-up data. Exclusion criteria included: (a) insufficient sample volume; (b) significant hemolysis or clotting; and (c) incomplete clinical data. This study was approved by the Institutional Ethics Committee of the First Affiliated Hospital of Nanjing Medical University (2022-SR-621), and informed consent was specifically waived by the ethics committee.

### HFC detection

All serous effusion samples were mixed by inversion and then analyzed using a Sysmex XN-10 hematology analyzer (Sysmex Corporation, Kobe, Japan) in BF mode. The analyzer employs semiconductor laser flow cytometry in combination with nucleic acid fluorescence staining to count and classify cells based on forward scatter (FSC), side scatter (SSC), and side fluorescence (SFL) signals. SFL primarily reflects the nucleic acid content in cells. Higher nucleic acid content results in stronger fluorescence signals. When the fluorescence intensity exceeded the the instrument-defined threshold, the cell was classified as a high-fluorescent cell and appeared above the white blood cells in the scatter plot. HFCs were reported as absolute number (HFC#) and percentage (HFC%), where HFC% was defined as the proportion of HFCs among white blood cells. The total nucleated cell count (TC) was calculated as the sum of WBC and HFC counts. The Sysmex XN-10 hematology analyzer is calibrated every six months in accordance with the requirements of ISO 15189:2022 (International Organization for Standardization ISO 15189:2022, 2022), and internal quality control is performed daily to ensure result reliability.

### Detection of other biomarkers

Serous effusion samples were centrifuged at 3,000 rpm for 5 min, and the supernatants were collected for subsequent biomarker analysis. CEA and cytokeratin 19 fragment (CYFRA21-1) levels were detected using a Cobas E602 electrochemiluminescence immunoassay analyzer (Roche, Basel, Switzerland). Lactate dehydrogenase (LDH) and adenosine deaminase (ADA) levels were detected using a Beckman Coulter AU5800 chemistry analyzer (Beckman Coulter, Brea, CA, USA). All procedures were performed according to the manufacturer’s instructions and laboratory standard operating procedures (SOP).

### Statistical analysis

Data analysis in this study was performed using SPSS 22.0 (SPSS, Inc., Armonk, NY, USA) and MedCalc 22.023 (MedCalc Software Ltd. Ostend, Belgium). Continuous variables were expressed as median and interquartile range (IQR). The Mann–Whitney U test was used for intergroup comparisons. A *P* value of less than 0.05 was considered statistically significant. Variables with *P* < 0.05 in the univariate analysis were further incorporated into the multivariate logistic regression analysis. The results of the multivariate logistic regression analysis are presented as odds ratio (OR) and 95% confidence interval (CI). Receiver operating characteristic (ROC) curve analysis was performed to evaluate the diagnostic performance of individual and combined biomarkers with the calculation of the area under the curve (AUC), sensitivity, specificity, and cutoff values. A *P* value less than 0.05 was considered statistically significant.

## Results

### Collected serous effusion samples

In this study, a total of 978 serous effusions samples were collected. Based on pathology results, imaging examinations and other clinical evidence, 520 were diagnosed as malignant effusions and 458 as benign effusions. Malignant effusions included 297 cases of lung cancer, 62 of gastric cancer, 31 of lymphoma, 29 of liver cancer, 18 of breast cancer, 17 of colon cancer, 17 of ovarian cancer, 10 of esophageal cancer, 10 of pancreatic cancer, six of cholangiocarcinoma, and 23 of other malignant tumors. Benign effusions comprised 342 cases of pulmonary and other infections, 77 of liver cirrhosis/liver failure, 23 of cardiac dysfunction, 13 of trauma and three of other conditions.

### Comparison of HFC and other biomarkers levels between the malignant and benign effusion groups

The comparisons of the HFC#, HFC%, TC, WBC, RBC, CEA, LDH, and ADA levels between the malignant and benign effusion groups are shown in Table 1. The statistical results revealed that the HFC#, HFC% and CEA levels were significantly higher in the malignant effusion group than in the benign group (all *P* < 0.001). Additionally, LDH (*P* = 0.004) and RBC (*P* = 0.048) levels were significantly elevated in the malignant group, whereas no significant differences were observed in TC (*P* = 0.354), WBC (*P* = 0.954), or ADA (*P* = 0.409) levels between the two groups. Variables with *P* < 0.05 in Table 1 were included in the multivariate analysis, and the statistical results are shown in Table 2. Multivariate logistic regression analysis identified high levels of HFC# (OR 1.002, 95% CI [1.000–1.003], *P* = 0.004), HFC% (OR 1.038, 95% CI [1.018–1.057], *P* < 0.001), CEA (OR 1.040, 95% CI [1.029–1.051], *P* < 0.001) and LDH (OR 1.000, 95% CI [0.999–1.000], *P* = 0.002) as independent predictors of malignant effusion. These results confirm that HFC# and HFC% are statistically independent factors associated with malignant effusion.

**Table 1:** Comparison of HFC and other biomarkers between the malignant and benign effusion groups.

Biomarkers	Malignant effusion group	Benign effusion group	*P* value
HFC# (/μL)	42 (16, 149)	13 (4, 38)	<0.001
HFC% (/100WBC)	6.4 (2.3, 15.8)	1.5 (0.4, 5.5)	<0.001
TC (10^6^/L)	950 (440, 2,081)	832 (235, 2,733)	0.354
WBC (10^6^/L)	842 (390, 1,814)	807 (231, 2,711)	0.954
RBC (10^6^/L)	4,000 (1,000, 22,000)	3,000 (1,000, 14,000)	0.048
CEA (ng/mL)	17.90 (1.93, 215.23)	1.22 (0.73, 2.05)	<0.001
LDH (U/L)	289 (167, 557)	230 (116, 575)	0.004
ADA (U/L)	10.9 (6.9, 14.7)	11.1 (7.3, 16.3)	0.409

**Notes.**

Data are expressed as the median and interquartile range (IQR).

**Table 2:** Multivariate logistic regression analysis for the prediction of malignant effusion.

Variable	OR (95% CI)	*P* value
HFC# (/μL)	1.002 (1.000–1.003)	0.004
HFC% (/100 WBC)	1.038 (1.018–1.057)	<0.001
CEA (ng/mL)	1.040 (1.029–1.051)	<0.001
LDH (U/L)	1.000 (0.999–1.000)	0.002
RBC (10^6^/L)	0.999 (0.999–1.000)	0.997

### Diagnostic performance of HFC in malignant effusions

**Figure 1:**
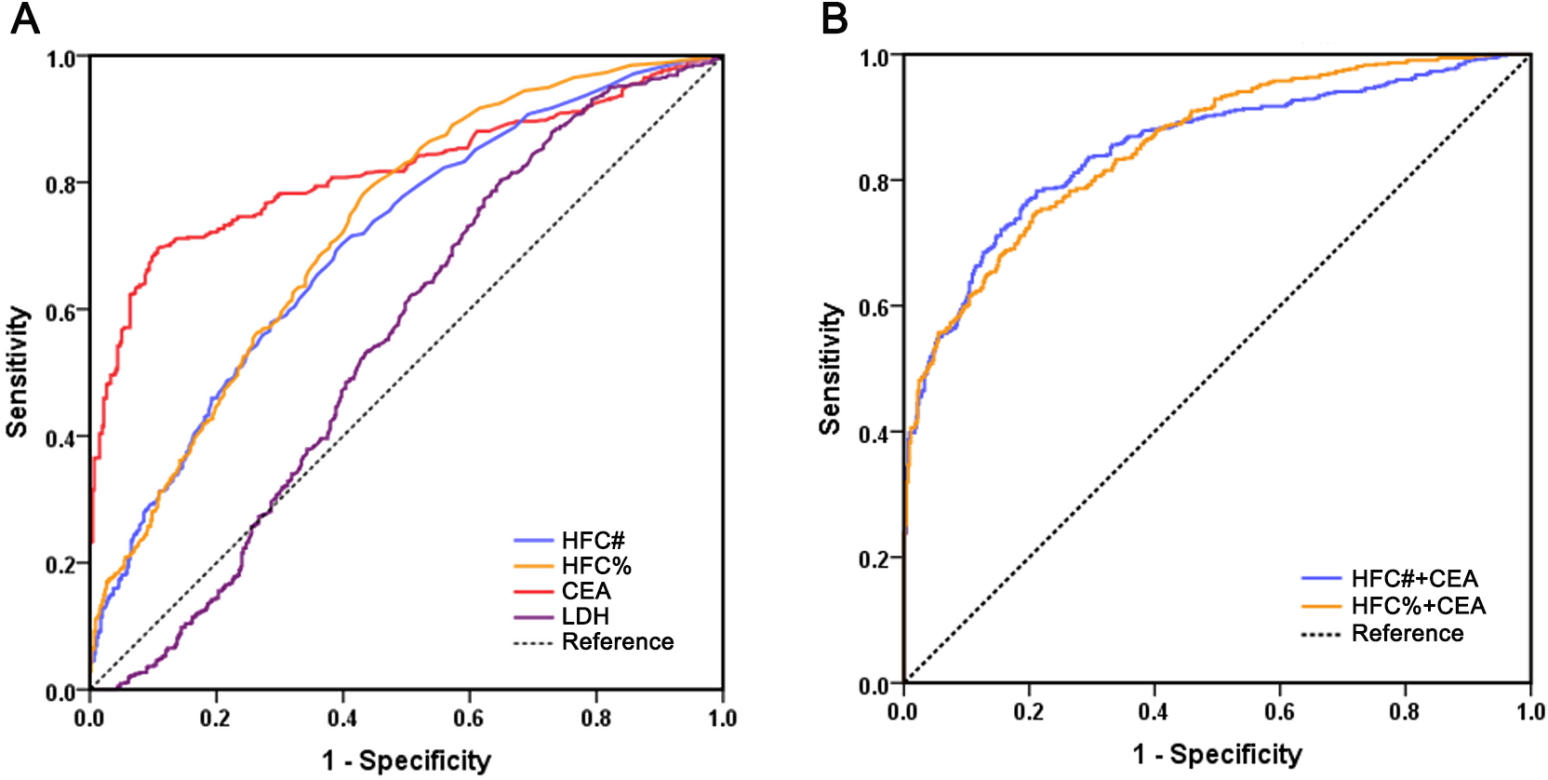
Comparison of receiver operating characteristics curves of HFC#, HFC%, CEA, LDH and multi-marker combinations for malignant effusion. (A) ROC curves of individual biomarkers HFC#, HFC%, CEA and LDH; (B) ROC curves of biomarker combinations.

To evaluate the diagnostic performance of HFC in malignant effusion and compare it with CEA and LDH, we performed ROC curve analysis (Fig. 1). The results revealed that CEA had the best diagnostic ability among all biomarkers with a sensitivity of 69.6% and specificity of 89.3% at a cutoff value of 2.9 ng/mL. The AUC of CEA was 0.817, which was significantly higher than HFC% (*P* < 0.001), HFC# (*P* < 0.001) and LDH (*P* < 0.001). HFC showed relatively high diagnostic ability for malignant effusions: HFC% at a cutoff of 2.1% demonstrated 78.3% sensitivity and 56.8% specificity, while HFC# at a cutoff of 20/µL showed 70.2% sensitivity and 60.3% specificity. The AUC values of HFC% (AUC 0.727, 95% CI [0.695–0.758], *P* < 0.001) and HFC# (AUC 0.703, 95% CI [0.671–0.736], *P* < 0.001) were both significantly higher than that of LDH (AUC 0.553, 95% CI [0.516–0.590]), with no significant difference between HFC% and HFC# (*P* = 0.207).

To further improve the diagnostic performance, different biomarkers were combined for evaluation (Table 3). The diagnostic performance of HFC% combined with CEA (AUC 0.855, 95% CI [0.832–0.878]) was significantly better than that of CEA alone (*P* = 0.003), with a sensitivity of 74.6% and specificity of 79.3%. Moreover, the combination of HFC# with CEA also significantly improved diagnostic performance compared with CEA alone (AUC 0.851, 95% CI [0.827–0.875], *P* = 0.001), yielding a sensitivity of 78.3% and a specificity of 78.8%. However, there was no significant difference in the AUCs beteeen HFC% combined with CEA and HFC# combined with CEA (*P* = 0.699). Therefore, compared with using individual indicators, the combination of either HFC% or HFC# with CEA significantly improved diagnostic performance.

**Table 3:** Diagnostic performance of HFC, CEA, and LDH for malignant effusion.

Biomarkers	AUC	95% CI	Cutoff value	Sensitivity (%)	Specificity (%)
HFC# (/μL)	0.703	0.671–0.736	20	70.2	60.3
HFC% (/100WBC)	0.727	0.695–0.758	2.1	78.3	56.8
CEA (ng/mL)	0.817	0.790–0.844	2.9	69.6	89.3
LDH (U/L)	0.553	0.516–0.590	146	80.4	35.2
HFC#+CEA	0.851	0.827–0.875		78.3	78.8
HFC%+CEA	0.855	0.832–0.878		74.6	79.3

### Diagnostic performance of HFC in malignant effusions associated with lung cancer

**Figure 2:**
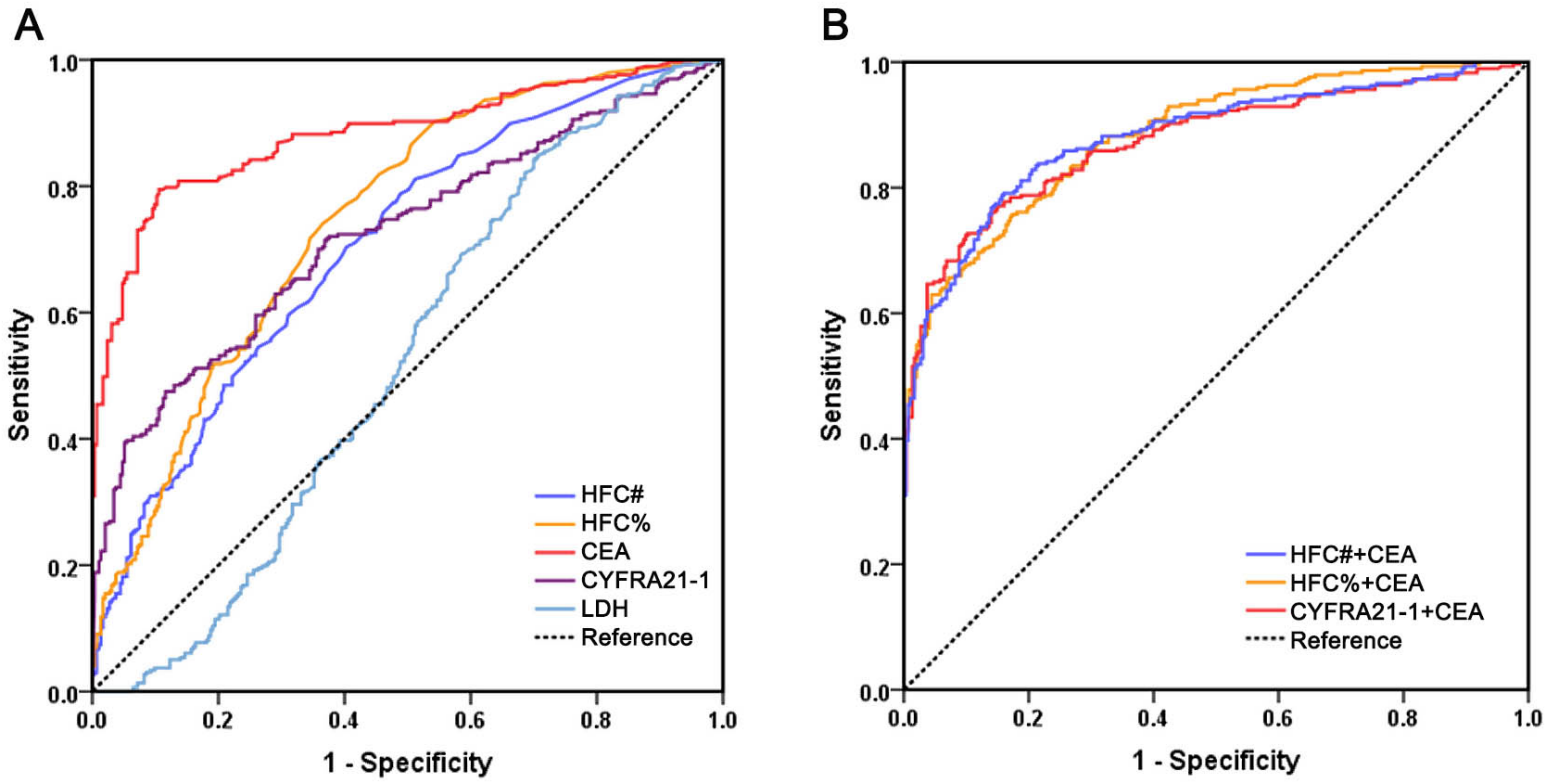
Comparison of receiver operating characteristics curves of HFC#, HFC%, CEA, CYFRA21-1, LDH and multi-marker combinations for malignant effusion associated with lung cancer. (A) ROC curves of individual biomarkers HFC#, HFC%, CEA, CYFRA21-1 and LDH; (B) ROC curves of biomarker combinations.

Given that lung cancer is one of the most common causes of malignant serous effusions and constituted the largest subgroup of malignancies in our cohort, we performed ROC analysis using data from 297 patients with lung cancer and 293 patients with lung infectious diseases to evaluate the diagnostic performance of HFC in lung cancer-associated malignant effusion (Fig. 2). We also included CYFRA 21-1, a conventional biomarker for lung cancer, for comparison. The results revealed that among individual biomarkers, CEA still exhibited the best diagnostic performance, with an AUC of 0.882, which was significantly higher than HFC% (AUC 0.746, 95% CI [0.707–0.785], *P* < 0.001), HFC# (AUC 0.706, 95% CI [0.665–0.747], *P* < 0.001) and CYFRA21-1 (AUC 0.724, 95% CI [0.683–0.764], *P* < 0.001) (Table 4). No significant differences were observed among HFC%, HFC# and CYFRA21-1 (HFC% *vs.* CYFRA21-1, *P* = 0.469; HFC# *vs.* CYFRA21-1, *P* = 0.621; HFC% *vs.* HFC#, *P* = 0.071). For biomarker combinations, neither the combination of HFC with CEA nor that of CYFRA 21-1 with CEA showed significantly better diagnostic performance than CEA alone, indicating that CEA retains an irreplaceable role in diagnosing malignant effusions associated with lung cancer.

**Table 4:** Diagnostic performance of HFC, CEA, CYFRA21-1, and LDH for malignant effusion associated with lung cancer.

Biomarkers	AUC	95% CI	Cutoff value	Sensitivity (%)	Specificity (%)
HFC# (/μL)	0.706	0.665–0.747	21	70.4	59.7
HFC% (/100WBC)	0.746	0.707–0.785	2.0	74.1	63.5
CEA (ng/mL)	0.882	0.854–0.910	3.1	79.5	89.4
CYFRA21-1 (ng/mL)	0.724	0.683–0.764	73.1	47.5	88.4
LDH (U/L)	0.523	0.475–0.570	166	84.5	29.7
HFC#+CEA	0.881	0.853–0.909		78.3	78.8
HFC%+CEA	0.884	0.858–0.910		74.6	79.3
Cyfra21-1+CEA	0.875	0.846–0.903		73.3	80.1

## Discussion

This study demonstrated that HFC has significant diagnostic value in the identification of malignant serous effusions. In particular, the combination of HFC with CEA significantly improved diagnostic performance. As a rapid and automated laboratory parameter, HFC shows considerable potential for clinical application.

In recent years, with the rapid development of liquid biopsy technology, an increasing number of studies have focused on the detection of tumor cells in serous effusions. For example, circulating tumor DNA (ctDNA) analysis, exosome profiling and single-cell sequencing have been progressively applied to the diagnosis of malignant effusions (Nikanjam, Kato & Kurzrock, 2022; Bao et al., 2024). ctDNA analysis can achieve early diagnosis of tumors by identifying tumor-specific gene mutations or epigenetic changes, but its sensitivity is limited by the DNA concentration in body fluids and tumor heterogeneity (Siravegna et al., 2017). Exosome analysis provides more comprehensive tumor information by extracting biomolecules such as proteins and RNA in vesicles secreted by tumor cells, yet the isolation and detection procedures remain complex, costly, and lack standardization (Zhang & Yu, 2019; Yu et al., 2022). Single-cell sequencing can characterize tumor cell subpopulations in body fluids and reveal molecular heterogeneity, but it is time-consuming, requires sophisticated equipment, and is currently confined to research settings, making large-scale clinical application challenging (Lei et al., 2021; Xu et al., 2022). Although these technologies show potential in scientific research, their clinical translation faces multiple challenges, including cost, standardization, and reproducibility.

Given these limitations, there is a clear need for more practical and accessible diagnostic tools. Our study demonstrates that HFC analysis effectively addresses this gap. Compared to conventional cytology, HFC analysis offers distinct advantages in speed and automation. Cytology examination is time-consuming and highly dependent on examiner expertise, resulting in variable sensitivity. A large meta-analysis indicated that although conventional cytology maintains high specificity (95%–99%) for malignant effusions, its sensitivity remains limited to 57%–77% (Ahuja et al., 2024). In contrast, HFC analysis on the Sysmex XN-10 provides automated and objective results within minutes during routine cell counting, thereby facilitating rapid screening. Moreover, previous studies have shown that by optimizing the HFC cutoff value, sensitivity for detecting malignant effusions can reach 87%–93% (Favresse et al., 2020; Cho et al., 2022).

The detection of HFC is based on the high nucleic acid content of tumor cells, which can effectively differentiate from normal cells through flow cytometry combined with fluorescence staining (Wong-Arteta et al., 2018; Sun et al., 2020). This study revealed that HFC level in malignant serous effusions was significantly higher than that in benign effusions. In addition to the commonly used tumor marker CEA, our study also incorporated conventional biochemical parameters for serous effusion analysis—LDH and ADA—as previous studies have reported their potential utility in identifying malignant effusions and providing prognostic information (Santotoribio, 2025). Multivariate analysis showed that HFC, CEA and LDH were all significant independent predictors of malignant effusion. However, in ROC analysis, LDH demonstrated poor diagnostic performance for malignant effusion, and HFC performed significantly better than LDH. Furthermore, among individual biomarkers, CEA exhibited the best diagnostic ability in differentiating malignant from benign serous effusions, consistent with the findings of multiple studies (Wu et al., 2019; Ai et al., 2024; Wang et al., 2023). Compared with CEA, however, HFC may hold particular significance in the diagnosis of malignant effusions due to its higher sensitivity and faster detection speed, albeit at the cost of lower specificity. Specifically, HFC analysis is integrated into the automated cell count procedure on the Sysmex XN-10 analyzer, providing results within minutes as part of the initial sample workup. In contrast, CEA measurement requires separate, batch-processed immunoassay testing, which typically takes several hours to complete. Based on the high nucleic acid fluorescence characteristics of tumor cells, HFC directly reflects cellular proliferation activity. CEA, as a glycoprotein biomarker, may be influenced by inflammation, benign lesions or other tumor types, leading to false-positive or false-negative results (Abouzid et al., 2024; Khan et al., 2025). In this study, the specificity of CEA was 89.3%, but its sensitivity was only 69.6%; in comparison, the sensitivity of HFC% was 78.3%. It is worth noting that most false positive HFC results are attributable to interference by mesothelial cells and macrophages (Xu et al., 2017). The high metabolic activity of these cells may produce fluorescence signals similar to those of malignant tumor cells during HFC detection. Therefore, high nucleic acid fluorescence signals in inflammation or reactive effusions, resembling those of tumor cells, may lead to instrument misclassification. Conversely, false negative results may occur in samples with low tumor cellularity, which fail to exceed the high fluorescence threshold. This observation suggests that differentiating benign from malignant serous effusions still requires integration with morphological examination or other tumor biomarkers. Therefore, HFC shows the most promise as a rapid screening tool to be used in conjunction with CEA measurement and conventional cytology.

Compared with other similar studies on malignant effusion diagnosis, which have typically involved more limited cohorts focused on specific clinical contexts such as liver cancer or lung cancer (Zhang et al., 2025; Wu et al., 2019; Wang et al., 2013), our study featured a substantially larger sample size and the inclusion of a diverse spectrum of tumor types, thereby enhancing the generalizability of our findings. This study systematically evaluated the diagnostic performance of the combination of HFC and CEA. The AUC of CEA combined with HFC was significantly higher than that of individual biomarkers, making an optimal clinical strategy for the rapid identification of malignant effusion. Moreover, we independently analyzed the diagnostic performance and cutoff value of HFC for malignant effusion associated with lung cancer. Although the diagnostic ability of HFC was second only to that of CEA, HFC showed no significant difference from CYFRA21-1, a conventional lung cancer biomarker. Therefore, while the diagnostic performance of HFC alone does not surpass that of CEA, its primary clinical value lies in its role within a combined model, as the integration of HFC with CEA demonstrated the highest diagnostic accuracy for identifying malignant effusions. However, this study has several limitations. First, the detection of HFCs depends on Sysmex series analyzers, and standardization across different laboratories still needs further investigation. Second, the applicability of the cutoff value of HFC in different tumor types needs further study, as its diagnostic performance may vary in some rare malignancies. Third, mesothelial cells and macrophages with high nucleic acid content may be detected as HFCs by the automated analyzer, potentially leading to false-positive results and affecting diagnostic accuracy. It should be clearly emphasized that the Sysmex XN-10 cannot distinguish or quantify mesothelial cells and macrophages separately from tumor cells—an inherent technical limitation of the current methodology. As accurate differentiation requires manual microscopic examination, futher detailed data on this aspect need to be supplemented in future validation studies.

## Conclusions

In conclusion, this study demonstrates that combining HFC with CEA offers meaningful clinical value for identifying malignant serous effusions. The combined use of both markers yields significantly improved diagnostic accuracy compared to either biomarker used alone. Thus, HFC used in conjunction with CEA may serve as a useful adjunct to cytomorphologic evaluation, enabling rapid clinical screening of malignant effusions.

##  Supplemental Information

10.7717/peerj.20528/supp-1 Supplemental Information 1 Raw data

## Additional Informations and Declarations

### Data Availability

The following information was supplied regarding data availability: The raw measurements are available in the Supplementary File.
